# The role of inflammation in pathogenesis of juvenile schizophrenia

**DOI:** 10.1192/j.eurpsy.2021.2024

**Published:** 2021-08-13

**Authors:** S. Zozulya, M. Omelchenko, I. Otman, A. Yakimets, Z. Sarmanova, V. Kaleda, T. Klyushnik

**Affiliations:** 1 Laboratory Of Neuroimmunology, Mental Health Research Centre, Moscow, Russian Federation; 2 Department Of Youth Psychiatry, Mental Health Research Centre, Moscow, Russian Federation

**Keywords:** attenuated symptoms of schizophrenic spectrum, first-episode psychosis, inflammatory markers, juvenile depression

## Abstract

**Introduction:**

Inflammation is now known to be a key factor in the development of schizophrenia. In this regard, the study of the pathogenic role of inflammation in the early stages of schizophrenic process is of particular importance, making it possible to assess its activity and to predict the development of the disease.

**Objectives:**

To compare the dynamics of inflammatory markers in blood of first-episode psychosis (FEP) patients and people at risk signs for schizophrenia in the course of the treatment. Juvenile depression (JD) with attenuated symptoms of schizophrenic spectrum (ASSS) was investigated as a risk group.

**Methods:**

The patients aged 17-25 years (20 people, of which 10 FEP patients (F20) and 10 JD with ASSS ones (F32.1-2, F32.38, F32.8)) were examined at admission to the hospital and at discharge. The controls consisted of 10 healthy volunteers. Symptom severity was collected using PANSS, SOPS, SANS, HDRS. The inflammation markers (TNF-α, IL-6, IL-10, leukocyte elastase (LE), CRP, α1-proteinase inhibitor (α1-PI), anti-S100-beta antibodies) were determined in blood.

**Results:**

An increase of inflammatory markers in both groups compared to controls was found (p<0,05). The highest values of IL-6, LE, CRP, α1-PI and anti-S100-beta antibodies in FEP patients were revealed (p=0,03). After the treatment, the positive trend of inflammatory markers in FEP patients (p<0,05), but not in JD with ASSS patients was detected (except LE activity, p<0,05).

**Conclusions:**

The results confirm the pathogenic role of inflammation in the development of endogenous mental disorders. The inflammatory markers studied reflect the activity of the pathological process in the early stages of schizophrenia.

**Disclosure:**

No significant relationships.

